# Underwater Low-Frequency Magnetic Field Detection Based on *Rao*’s Sliding Threshold Method

**DOI:** 10.3390/s25113364

**Published:** 2025-05-27

**Authors:** Yi Li, Jiawei Zhang

**Affiliations:** Naval University of Engineering, Wuhan 430030, China; aoyi999666@126.com

**Keywords:** ship, axial frequency magnetic field, *Rao* detector, non-Gaussian noise, sliding threshold

## Abstract

This paper proposes a joint time–frequency analysis method that combines *Rao* detector with dynamic sliding thresholds to enhance the detection performance of electric source axial frequency magnetic field signals. For each signal-to-noise ratio (SNR) point, 1000 Monte Carlo simulations were independently conducted, with SNR ranging from 15 dB to −30 dB. The results show that the proposed method maintains high detection rates even at extremely low SNRs, achieving about 90% detection probability at −13 dB, significantly outperforming traditional energy detectors (with a threshold of 2 dB). Under conditions where the detection probability is ≥90% and the false alarm probability is 10^−3^, the SNR threshold for the *Rao* detector is reduced by 15 dB compared to energy detectors, greatly improving detection performance. Even at lower SNRs (−30 dB), the *Rao* detector still maintains a certain detection rate, while the detection rate of energy detectors rapidly drops to zero. Further analysis of the impact of different frequencies (1–5 Hz) and CPA distances (45–80 cm) on performance verifies the algorithm’s robustness and practicality in complex non-Gaussian noise environments. This method provides an effective technical solution for low SNR detection of ship axial frequency magnetic fields and has good potential for practical application.

## 1. Introduction

In classical physical field theory, electromagnetic fields, acoustic fields, and thermal fields are the core physical quantities for underwater target detection. Among these, shaft-frequency electromagnetic fields (SREMs) have become an important research direction in non-acoustic underwater target detection due to their unique time–frequency modulation characteristics [1,2,3,4]. Shaft-frequency electromagnetic fields are generated by the modulation of corrosion currents and cathodic protection currents by ship propeller rotation. Their fundamental frequency typically ranges from 0.5 to 30 Hz, and the signal strength decays slowly with distance, allowing a detection radius of over 600 m [5,6].

Current research predominantly focuses on shaft-rate electric fields, while studies on detection methods for shaft-rate magnetic fields remain limited [7]. Traditional underwater magnetic field detection methods (e.g., energy detection, power spectrum detection) exhibit significant performance bottlenecks in non-Gaussian noise and low signal-to-noise ratio (SNR) environments.

As the optimal detector under Gaussian noise, energy detection typically relies on accurate noise power estimation for threshold setting. However, in non-Gaussian noise (e.g., mixed Gaussian distribution with kurtosis γ ≠ 0), its detection statistics substantially deviate from theoretical optima. For instance, the authors of [4] demonstrate that when noise kurtosis γ = 0.5 (moderate non-Gaussianity), the SNR threshold (defined as the minimum SNR achieving detection probability Pd = 0.9) reaches −5 dB for energy detection. In practical marine environments where noise kurtosis commonly ranges from γ = −0.5 to 0.5, energy detection becomes virtually ineffective when SNR falls below −3 dB.

Sliding power spectral detection enhances the signal-to-noise ratio through frequency domain accumulation, but fundamentally still depends on the Gaussian noise assumption. Field measurements reveal that when SNR drops below −8 dB, its false alarm rate surges by over 10-fold.

The core limitation of these methods lies in their inadequate modeling of both the non-Gaussian noise characteristics and the time–frequency modulation features of signals, rendering them incapable of effectively distinguishing target signals from noise in low-SNR scenarios. 

Statistical Deviation Caused by Non-Gaussian Noise:

The impulsive nature of non-Gaussian noise (e.g., dual-variance components in Gaussian mixture models) amplifies outliers through the squaring operation in energy detectors, leading to uncontrolled false alarm rates. For instance, when noise follows a Gaussian mixture distribution with variances σ_1_^2^ = 1 (80% occurrence) and σ_2_^2^ = 20 (20% occurrence), the energy detector’s false alarm rate increases by 2–3 orders of magnitude compared to Gaussian noise scenarios.

Insufficient Utilization of Signal Characteristics:

Ship shaft-rate magnetic fields exhibit harmonic-rich time–frequency structures (fundamental frequency: 1–7 Hz, harmonics up to 21 Hz). Conventional methods only exploit total signal energy while neglecting phase and amplitude modulation information across frequency components. For example:

At SNR = −12 dB, energy detection achieves merely 0.3 detection probability.

Stochastic resonance-based techniques can enhance signals but require a priori parameter configuration [8].

As underwater detection technology advances, electrical source axial frequency magnetic field detection faces new challenges. Although current research on magnetic field detection has made some progress in noise suppression and algorithm optimization, achieving stable and reliable axial frequency magnetic field detection remains a significant challenge in complex environments with low signal-to-noise ratios and non-Gaussian noise dominance. In existing studies, some papers focus on improving signal quality through filtering algorithms, such as [9], which uses specific filtering algorithms to process magnetic field signals but lacks the sufficient characterization of the complex properties of non-Gaussian noise; [10] outlines detection based on traditional models without fully integrating the characteristics of the signal source and noise statistics. Overall, most studies emphasize changes in signal energy or general filtering processing, with insufficient exploration of the signal model and noise model characteristics of electrical source axial frequency magnetic fields, making it difficult to fully utilize the effective information contained in the target [11,12].

In recent years, deep learning models such as convolutional neural networks (CNNs) and variational autoencoders (VAEs) have been widely applied for marine noise suppression. For instance, CNNs demonstrate exceptional feature extraction capabilities in non-Gaussian noise environments, significantly improving detection accuracy for weak signals. Conditional variational autoencoders (CVAEs) effectively eliminate shallow-water impulsive noise by learning nonlinear mapping relationships between noise and clean signals. However, these methods rely on extensive labeled training data and exhibit high computational complexity, making them difficult to deploy in real-time scenarios with limited hardware resources [13].

Adaptive filtering algorithms (e.g., recursive least squares, RLS; Kalman filters) excel in tracking time-varying noise. The RLS algorithm demonstrates fast convergence and low computational cost in noise cancelation tasks, while adaptive Kalman filters maintain high target detection accuracy in complex sea conditions through dynamic parameter adjustment. Nevertheless, these methods heavily depend on accurate noise statistics and system modeling, potentially failing in mixed Gaussian/non-Gaussian noise environments [14].

Hybrid approaches combining traditional signal processing with machine learning are emerging. For example, the MTCEEMD-Volterra adaptive filtering framework integrates empirical mode decomposition with adaptive filtering to suppress sea clutter while maintaining computational efficiency. Though multi-sensor fusion (e.g., radar and infrared imaging) enhances detection robustness, it requires additional hardware and calibration, increasing engineering complexity [15].

To address these challenges, this paper proposes a sliding-threshold detection method based on the *Rao* detector. The approach involves the following:

(1) Establishing an electrical source shaft-rate magnetic field signal model;
(2) Conducting in-depth analysis of non-Gaussian noise characteristics in surface platform measurement scenarios;
(3) Implementing noise modeling using Gaussian mixture models with real-time parameter estimation via expectation–maximization (EM) iteration [13];
(4) Calculating *Rao* test statistics and employing their moving average as a dynamic detection threshold for precise shaft-rate magnetic field detection.

The method’s effectiveness is verified through simulation experiments comparing *Rao* detection versus conventional energy detection performance and field data evaluations against typical magnetic detection methods, thus demonstrating superior non-Gaussian noise suppression and detection capability.

## 2. Modeling

In the presence of Gaussian background noise and without any prior information about the signal, the energy detector is known as an optimal detector due to its solid theoretical foundation and straightforward implementation [14]. This optimality derives from the statistical properties of Gaussian noise, allowing optimal threshold setting based on the energy statistic. However, in practical scenarios, especially in detecting axial magnetic fields from electrical sources, background noise often exhibits non-Gaussian characteristics such as impulsive components and heavy-tailed distributions. These non-Gaussian properties cause the performance of the energy detector to degrade progressively. For instance, in nearshore marine magnetic environments, noise is influenced by natural geomagnetic disturbances and anthropogenic electromagnetic interference, resulting in significant non-Gaussianity [15].

To address such challenging noise, the generalized likelihood ratio test (GLRT) can achieve improved detection by jointly estimating unknown parameters under both hypotheses (signal absence and presence). Nonetheless, the GLRT requires parameter estimation under both hypotheses, leading to high computational complexity. The *Rao* detection method, as an improved variant of the GLRT, estimates parameters only under the null hypothesis (no signal), greatly reducing computational burden while maintaining good detection performance in non-Gaussian noise, making it widely used in electromagnetic detection applications [16].

To detect the axial frequency magnetic field using the *Rao* detector, we establish the corresponding signal and noise models. The specific content is as follows:

### 2.1. Signal Model

The axial frequency magnetic field of the electrical source is modulated by the ship’s electrical source-related systems (such as corrosion protection devices, power supply circuits, etc.) as they rotate with the propeller, forming an extremely low-frequency magnetic field signal with the propeller speed as the fundamental frequency (typically ranging from 1 to 7 Hz). This signal has a zero mean characteristic; although its frequency range can be estimated based on the ship’s dynamic parameters, the specific frequency and amplitude remain unknown, and it contains rich harmonic components. Based on electromagnetic radiation theory and Fourier series expansion, the axial frequency magnetic field signal at the observation point can be expressed as [3]

(1) $$\vec{B}[n]=\sum_{k=1}^{M}(\vec{A_{k}}\cos(2\pi kf_{0}n)+\vec{B_{k}}\sin(2\pi kf_{0}n)),n=0,1,\cdots ,N-1$$

In Equation (1): B[n] represents the magnetic field vector of the electrical source axis at time n (excluding noise); M is the number of harmonic components, which is generally taken as M = 3 based on the actual distribution of harmonic energy detected. Specifically, through the spectral analysis of the frequency magnetic field signals from the electric source axis, we found that the first three harmonic components account for more than 90% of the total signal energy. The energy of subsequent higher-order harmonics is lower and their contribution to overall detection performance is limited. Additionally, choosing M = 3 ensures model accuracy and expressiveness while avoiding the computational burden of overly complex models, making it suitable for real-time online detection needs. Reference [5] also supports modeling signals based on the first three harmonics. A_k_ and B_k_ are the amplitude vectors of the kth harmonic; ω is the fundamental frequency, with f_0_ ∈ [fmin, fmax], where fmin and fmax are estimated from the operational parameters of the ship’s electrical source system, typically f_0_ ∈ [1, 7] Hz; n is the discrete time; and N is the total duration of the signal. For ease of subsequent analysis and calculation, it is converted into matrix form [5]:

(2) $$\left[\begin{array}{c} B_{x}[0]\\ B_{x}[1]\\ \vdots \\ B_{x}[N-1]\\ B_{y}[0]\\ B_{y}[1]\\ \vdots \\ B_{y}[N-1]\\ B_{z}[0]\\ B_{z}[1]\\ \vdots \\ B_{z}[N-1] \end{array}\right]=\left[\begin{array}{cccccc} C_{1} & S_{1} & C_{2} & S_{2} & C_{3} & S_{3} \end{array}\right]\left[\begin{array}{c} {\vec{A}}_{1}\\ {\vec{B}}_{1}\\ {\vec{A}}_{2}\\ {\vec{B}}_{2}\\ {\vec{A}}_{3}\\ {\vec{B}}_{3} \end{array}\right]$$

where among them, B_x_, B_y_, and B_z_ are the components of the electric source axis $\cos(2\pi kf_{0}n),\;(\sin(2\pi kf_{0}n)$ frequency magnetic field in three-dimensional space C_k_ and S_k_ are the coefficient matrices formed by them, which are calculated and generated based on discrete moment n and fundamental frequency f_0_.

### 2.2. Noise Model

When conducting electrical source axis-frequency magnetic field detection in marine environments, the long-term statistical characteristics of environmental noise during periods without targets often fail to meet the Gaussian distribution. Although the frequency range of axis-frequency magnetic fields (typically 1–7 Hz) is relatively clear, the Gaussian nature of environmental $w_{n}[n],n=1,2,\cdots ,N$, noise is often disrupted by the complex marine environment (such as seabed geological activities, artificial electromagnetic interference, etc.). Based on the non-Gaussianity study methods of noise from the literature [1], forzero-mean noise characterized by its probability density function (PDF) noise, and its non-Gaussian degree is characterized by the kurtosis relative to a Gaussian PDF, as shown in Equation (3):

(3) $$\gamma =\frac{E\left(w_{n}^{4}[n]\right)}{E^{2}\left(w_{n}^{2}[n]\right)}-3$$

E(·): represents the mean in Equation (3). Gaussian noise corresponds to 0, while non-Gaussian noise deviates from 0. Selected real-measured marine environmental magnetic field data was utilized for evaluation. Using magnetic field data measured by a surface floating platform, each data segment is 50 s long, with a total of 11 datasets covering the entire day’s environmental magnetic field conditions. The calculation results show that the noise kurtosis ranges from −0.5436 to 0.6009, significantly deviating from zero in most cases, indicating that the marine environmental magnetic field noise has non-Gaussian characteristics [4]. The Kolmogorov–Smirnov test verifies that the second-order zero-mean mixed Gaussian model has a fitting *p*-value greater than 0.05 and a fitting error less than 5% for all samples, while the single Gaussian model is rejected (*p* < 0.01). Therefore, the second-order zero-mean mixed Gaussian model can be used for fitting. The mixed Gaussian model simulates the non-Gaussian noise distribution through mixing coefficients, with its probability density function being

(4) $$P(w[n])=\frac{1-\varepsilon }{\sqrt{2\pi }\sigma_{1}}\exp\left(-\frac{w^{2}[n]}{2\sigma_{1}^{2}}\right)+\frac{\varepsilon }{\sqrt{2\pi }\sigma_{2}}\exp\left(-\frac{w^{2}[n]}{2\sigma_{2}^{2}}\right)$$

The 4th moment of the mixed Gaussian distribution is

(5) $$E\left(w^{4}[n]\right)=3(1-\varepsilon )\sigma_{1}^{4}+3\varepsilon \sigma_{2}^{4}$$

The peak value of mixed Gaussian noise can be obtained by substituting γ into Equation (5):

(6) $$\gamma =\frac{3\varepsilon (1-\varepsilon ){\left(\sigma_{1}^{2}-\sigma_{2}^{2}\right)}^{2}}{{\left[(1-\varepsilon )\sigma_{1}^{2}+\varepsilon \sigma_{2}^{2}\right]}^{2}}$$

### 2.3. Detection Method Implementation

Based on the signal model in Section 2.1 and the noise model analysis in Section 2.2, the complete data model is as follows:

(7) x[n]=s[n]+w[n]

x[n] represents the electric source axial frequency magnetic field signal (including noise) in Equation (7).

The following test statistics are defined:

(8) $$T_{Rao}[y]=\underset{f_{0}}{max}\;\frac{y^{H}H\left(f_{0}\right){\left(H^{H}\left(f_{0}\right)i(A)H\left(f_{0}\right)\right)}^{-1}H^{H}\left(f_{0}\right)y}{i(A)}$$

In Equation (8), max(·) represents the maximum value of within the range $f_{0}\in \left[f_{min},f_{max}\right]$. In the actual calculation, $f_{0}$ needs to be discretized into a series of frequency points (in the subsequent text, the frequency interval is set to 0.1 Hz, $y=[y[0],y[1],\cdots ,y[n],\cdots ,y[N-1]{]}^{T},y[n]=g(x[n])$. The derivation of this statistic is detailed in reference [4], with its core idea being to maximize the gradient of the log-likelihood ratio of the signal in mixed Gaussian noise using the *Rao* detection criterion. For Gaussian filtering operators, when used in real-time, their physical significance is equivalent to a limiter, and its form is g(·).

(9) $$g(w)=-\frac{\mathrm{dln}\;P(w)}{dw}=-\frac{dP(w)/dw}{P(w)}$$

The P(w) is the probability density function (PDF) of non-Gaussian noise, and ω represents zero-mean mixed Gaussian noise; A is the corresponding parameter value under condition H1; and the i(A) is information matrix factor, which is related to the PDF of the noise. For the mixed Gaussian distributed noise, it is also related to the mixing coefficient ω and variance $\sigma_{1}^{2},\sigma_{2}^{2}$ [1].

(10) $$i(A)=\int_{-\infty }^{\infty }\;\frac{(dP(w)/dw{)}^{2}}{P(w)}dw$$

Substitute g(w) into Equation i(A),

We thus obtain

(11) $$i(A)=\int_{-\infty }^{\infty }\;\frac{[-g(w)P(w){]}^{2}}{P(w)}dw=E\left[g(w{)}^{2}\right]\approx \frac{1}{N}\sum_{n=0}^{N-1}\;(y[n]{)}^{2}$$

It can be seen from the above Equation that $i\left(A\right)$ can be replaced by the mathematical expectation value of $g(w{)}^{2}$, and under the condition of no target, the mathematical average of the Gaussian filtering result can be used to approximate describe i(A), thus greatly reducing the calculation complexity.

In addition, when the fundamental frequency $f_{0}\geq 1/N$ (which can be achieved by adjusting the data length), the column vectors of $H\left(f_{0}\right)$ are approximately orthogonal.

(12) $$H^{H}\left(f_{0}\right)H\left(f_{0}\right)\approx (N/2)I$$

I is the identity matrix, then the detection statistic can be simplified to the detection, then the detection statistic can be simplified as

(13) $$T_{Rao}[y]={max}_{f_{0}}\;\frac{2}{i(A)N}y^{H}H\left(f_{0}\right)H^{H}\left(f_{0}\right)y$$

Due to

(14) $$H^{H}\left(f_{0}\right)y=\left[\begin{array}{l} \sum_{n=0}^{N-1}\;y[n]cos\left(2\pi f_{0}n\right)\\ \sum_{n=0}^{N-1}\;y[n]cos\left(4\pi f_{0}n\right)\\ \sum_{n=0}^{N-1}\;y[n]cos\left(6\pi f_{0}n\right)\\ \sum_{n=0}^{N=1}\;y[n]sin\left(2\pi f_{0}n\right)\\ \sum_{n=0}^{N=1}\;y[n]sin\left(4\pi f_{0}n\right)\\ \sum_{n=0}^{N-1}\;y[n]sin\left(6\pi f_{0}n\right) \end{array}\right]$$

The test statistics can be further simplified as

(15) $$T_{Rao\;}[y]={max}_{f_{0}}\;\frac{2}{i(A)N}\sum_{n=0}^{N-1}\;{\left|\sum_{k=1}^{M}\;y[n]\exp\;\left(-j2\pi kf_{0}n\right)\right|}^{2}$$

Thus, the detection y[n] statistics of the electric source axis frequency magnetic field can be obtained, and then the sliding threshold detection based on *Rao* detector can be realized.

### 2.4. Implementation Process

The steps for the magnetic field detection method based on *Rao* detector are as follows: First, estimate the parameters of the mixed Gaussian noise model under the assumption of H0 (no target). Then, solve the detection statistic $T_{Rao\;}$. To reduce the false alarm rate, a sliding threshold method is used for target detection, thereby improving the detection probability while maintaining a low false alarm probability. Additionally, when modeling environmental noise using the mixed Gaussian model (CM), due to the complexity of environmental noise characteristics, adaptive improvements need to be made to the *Rao* detection method [17]. First, estimate the PDF of environmental noise in real-time, which means estimating the parameters of the second-order zero mean mixed Gaussian model in real-time. Estimating the CM parameters requires only a few iterations (low computational cost), making this method computationally efficient. For the estimation of the second-order zero mean mixed Gaussian CM parameters in this paper, the EM iterative algorithm is used. Assuming that the statistical properties of the noise remain constant over a certain period, the noise of each sample during this period is denoted as $w_{n}[i]$, When the sample comes from the first Gaussian source $s_{n}[i]=1$, otherwise $s_{n}[i]=0$. The total number of Gaussian sources is $M_{s}=2$. According to Equation (16), calculate the expectation value of, i.e., the posterior probability that a sample comes from the qth Gaussian source [18]:

(16) $${\overset{ˆ}{s}}_{n}[i]=\frac{f\left(w_{n}[i]|\sigma_{q}^{2}\right)}{\sum_{j=1}^{2}\;\;f\left(w_{n}[i]|\sigma_{j}^{2}\right)}$$

where $\sigma_{q}^{2}$ is the noise variance:

(17) $$\sigma_{q}^{2}f\left(w_{n}[i]|\sigma_{q}^{2}\right)=\frac{1}{\sqrt{2\pi \sigma_{q}^{2}}}\exp\;\left(-\frac{w_{n}^{2}[i]}{2\sigma_{q}^{2}}\right)$$

Then, calculate the expected ${\overset{ˆ}{s}}_{n}[i]$ value of noise variance and mixing coefficient:

(18) $$\sigma_{q}^{2}=\frac{\sum_{i=1}^{n}\;\;{\overset{ˆ}{s}}_{n}[i]w_{n}^{2}[i]}{\sum_{i=1}^{n}\;\;{\overset{ˆ}{s}}_{n}[i]}\varepsilon =\frac{1}{n}\sum_{i=1}^{n}\;{\overset{ˆ}{s}}_{n}[i]$$

Thus, the iterative process is as follows: First, according to the experience, set $\sigma_{1}^{2}=1,\sigma_{2}^{2}=0.1\left(\sigma_{1}^{2}>\sigma_{2}^{2}\right),\varepsilon =0.5$; calculate the variance of the first Gaussian source as $\sigma_{1}^{2}=\frac{\sum_{i=1}^{n}\;\;\left(1-{\overset{ˆ}{s}}_{n}[i]\right)w_{n}^{2}[i]}{\sum_{i=1}^{n}\;\;\left(1-{\overset{ˆ}{s}}_{n}[i]\right)}$; then use Equation (16) to estimate ${\overset{ˆ}{s}}_{n}[i]$ and update $\sigma_{2}^{2}$, and the iterative termination condition is that the change in σ is less than the set value (of$\;10^{-2}$ order).

It should be noted that when estimating the CM parameters, the selected Δt samples should be the environmental magnetic field data from the moment before the moment to achieve adaptive CM parameter estimation and improve the detection probability.

The measured signal is first subjected to bandpass filtering (0.5–30 Hz) and environmental magnetic field mean compensation to obtain the target signal. The detection statistic *T_Rao_* is calculated by Equation (15). Calculate the average of the previous M *T_Rao_* values as T_mean_, then multiply T_mea_ by the coefficient α to determine the threshold value for the current moment. The threshold F[α] is

(19) $$F[\alpha ]=\alpha \cdot \frac{1}{M}\sum_{i=1}^{M}\;T_{Rao\;}[i]$$

Due to the significant increase in the data truncation process *T_Rao_*, after a short time Δt, the threshold F will exceed the actual environmental noise threshold, leading to misjudgment. Therefore, after detecting the target, confirm *T_Rao_*, which corresponds to the detection moment, as the upper limit of threshold F for this stage, reducing the probability of missed detections. This method works well when there is no target or only a weak target present [19].

## 3. Simulation Analysis

To evaluate the advantages of the electrical source axis frequency magnetic field detection method based on *Rao* detector over energy detection $sf_{0}N$ methods, the following simulation experiment was conducted. The fundamental frequency of the electrical source axis frequency magnetic field signal is set to 3 Hz, and it includes specific harmonic components. Each data segment is 500 samples long (5 s of detection data at an analog sampling rate of 6 Hz), with a total of 1000 simulations. Note that this simulation is solely for evaluating the effectiveness of the *Rao* detection method itself and is independent of the sliding threshold, which is set according to actual application requirements.

Since the probability density function (PDF) of the axial magnetic field signal and noise of the electrical source is known exactly during simulation, the optimal detection effect can be obtained by using the Neyman–Pearson (NP) criterion. Taking the NP upper bound as the reference, the detection statistic is

(20) $$T_{NP}(x)=\sum_{n=0}^{N-1}\;(|x[n]|-|x[n]-s[n]|)$$

where x is the magnetic field signal of the electrical source axis to be detected in Equation (20). In addition, for comparative analysis, the effect based on energy detection is obtained, and its detection statistic is

(21) $$T_{ED}(x)=\sum_{n=0}^{N-1}\;x^{2}[n]$$

## 4. The Experimental Part

To verify the effectiveness of the sliding threshold detection method based on the *Rao* detector for ship shaft frequency magnetic field detection, tests were conducted in a laboratory water tank using an electric source shaft frequency magnetic field generator. The sensors were fixed on a moving track, while the electric source shaft frequency magnetic field was arranged in a shallow water tank. The water tank was mixed to have electromagnetic properties that closely match those of the marine environment, better simulating the propagation process of electromagnetic signals from ocean targets in seawater. In the experiment, the electric source shaft frequency magnetic field current was set to 5 A, and the generator produced shaft frequency magnetic field signals at different frequencies of 1 Hz, 2 Hz, 3 Hz, and 5 Hz. Data collection was carried out at two CPA distances (closest point of approach) of 45 cm and 80 cm. Some conditions were repeated experiments (specific repeat counts are shown in the data statistics table mentioned earlier). High-precision magnetic field sensors were used to collect magnetic field signals under different frequencies and CPA distance conditions, with detailed records of signal frequency, CPA distance, and current being kept during each data collection.

**Figure 1:**
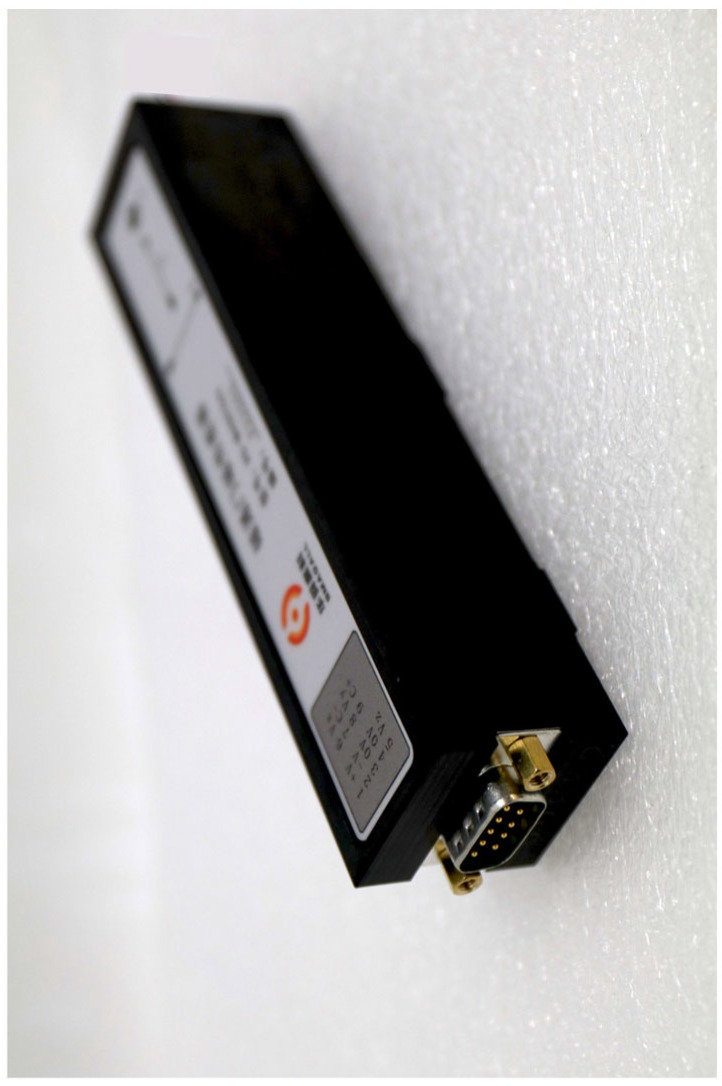
Three-axis fluxgate sensor.

**Figure 2:**
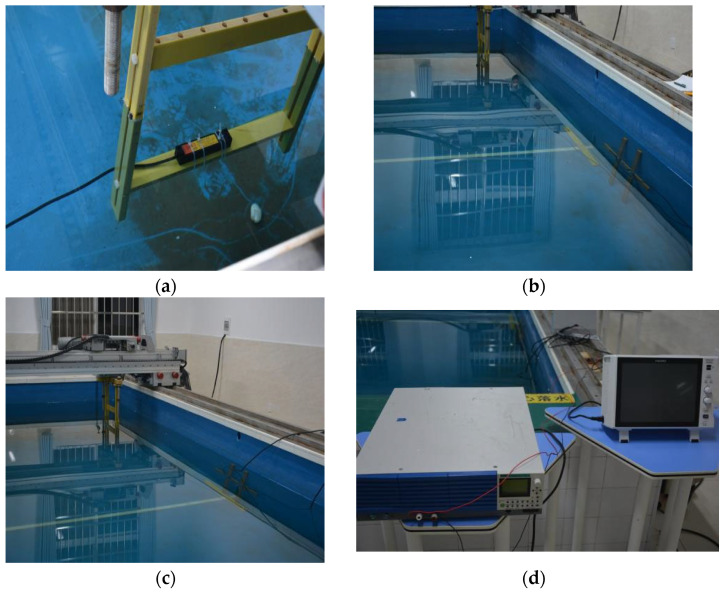
Layout of the experimental site: (**a**) sensor layout; (**b**) electric source magnetic field source layout; (**c**) measure the track layout; (**d**) upper monitor.

To accurately capture the subtle changes in magnetic fields required for the experiment, we selected the orthogonal standard series standard version tri-axis fluxgate sensor (model: HSF123-2H3-DZA). Its excellent sensitivity and low noise performance, along with minimal tri-axis consistency error, ensure the reliability of multi-channel measurement data. As shown in Figure 1 below, the product specifications are as follows: measurement range ±100 µT, tri-axis consistency error ≤0.3%, frequency domain noise ≤10 pTrms/√Hz at 1 Hz, time domain noise ≤0.1 nT RMS at 10 points per second, zero-point error ≤50 nT, and after correction by the sensitivity coefficient and zero-point offset coefficient, the absolute accuracy of the total field value does not exceed ±40 nT (see Figure 2).

**Figure 3:**
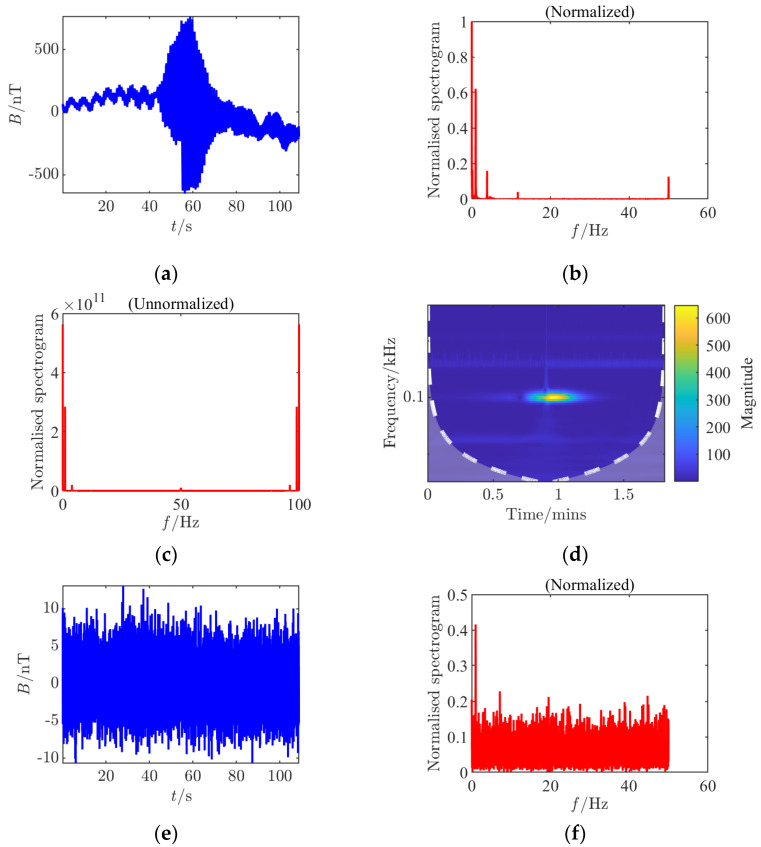

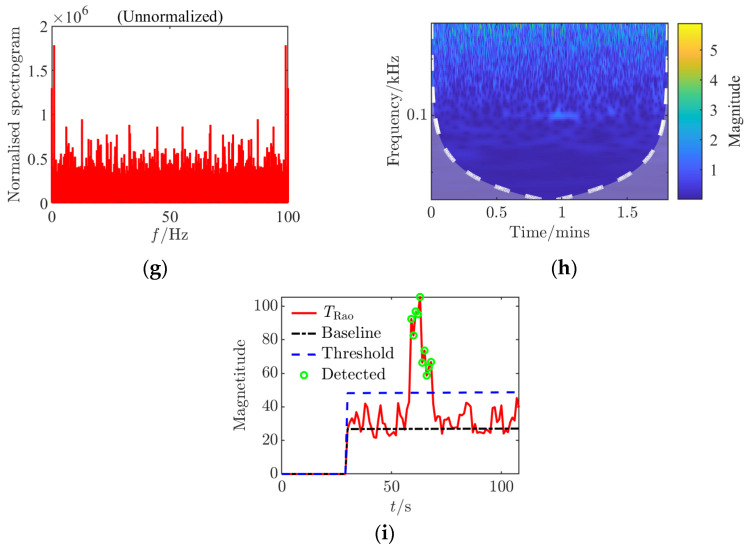
Time–frequency characteristics and detection results of Condition 1 at the signal detection threshold. (**a**) Time domain waveform; (**b**) normalized frequency spectrum of the clean signal; (**c**) raw frequency spectrum after noise removal; (**d**) continuous wavelet transform; (**e**) time domain waveform after noise addition; (**f**) normalized noisy frequency spectrum; (**g**) raw frequency spectrum with noise; (**h**) continuous wavelet transform after noise addition; (**i**) *Rao* test results.

**Figure 4:**
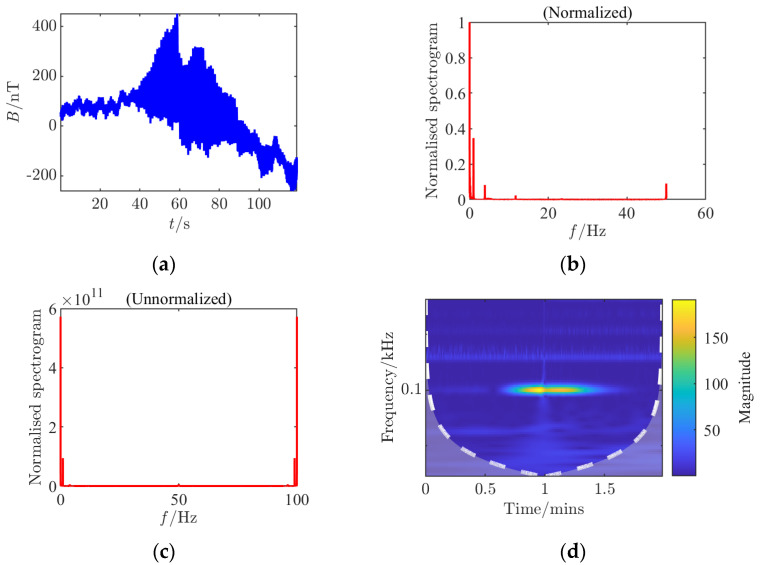

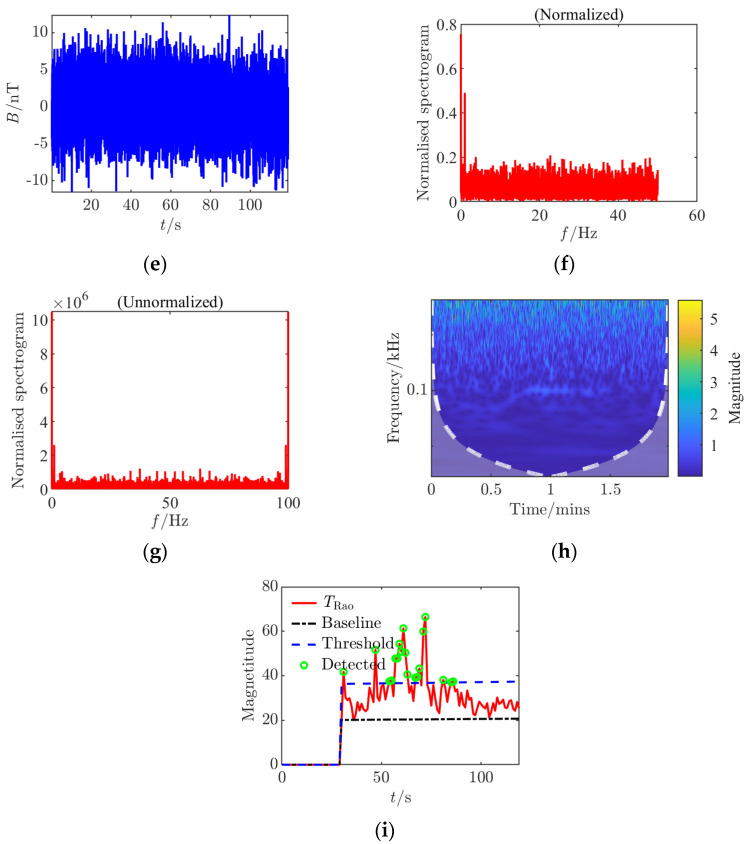
Time–frequency characteristics and detection results of Condition 2 at the signal detection threshold. (**a**) Time domain waveform, (**b**) normalized frequency spectrum of the clean signal; (**c**) raw frequency spectrum after noise removal; (**d**) continuous wavelet transform; (**e**) time domain waveform after noise addition; (**f**) normalized noisy frequency spectrum; (**g**) raw frequency spectrum with noise; (**h**) continuous wavelet transform after noise addition; (**i**) *Rao* test results.

**Figure 5:**
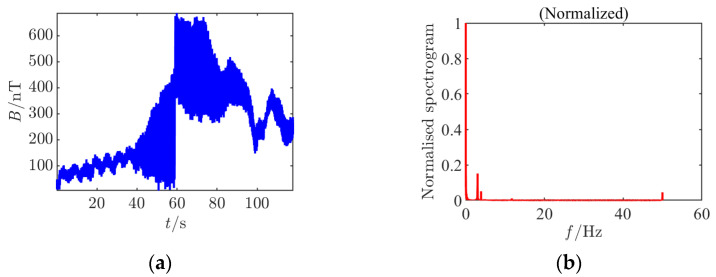

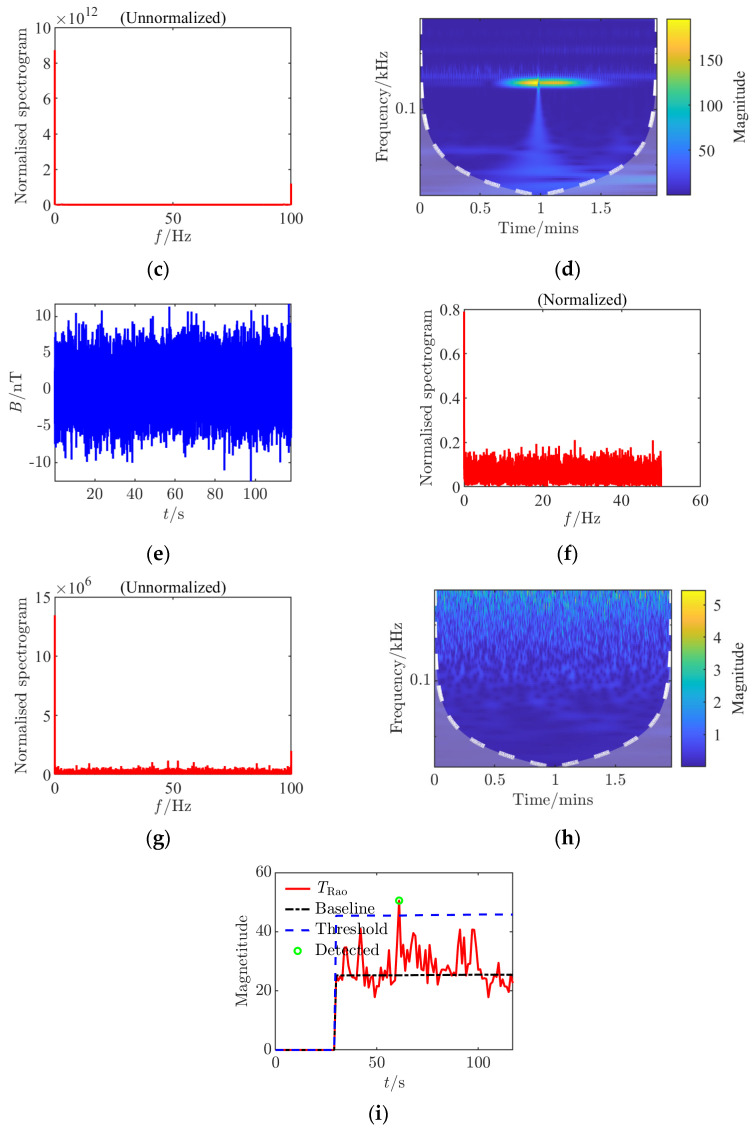
Time–frequency characteristics and detection results of Condition 3 at the signal detection threshold. (**a**) Time domain waveform; (**b**) normalized frequency spectrum of the clean signal; (**c**) raw frequency spectrum after noise removal; (**d**) continuous wavelet transform; (**e**) time domain waveform after noise addition; (**f**) normalized noisy frequency spectrum; (**g**) raw frequency spectrum with noise; (**h**) continuous wavelet transform after noise addition; (**i**) *Rao* test results.

**Figure 6:**
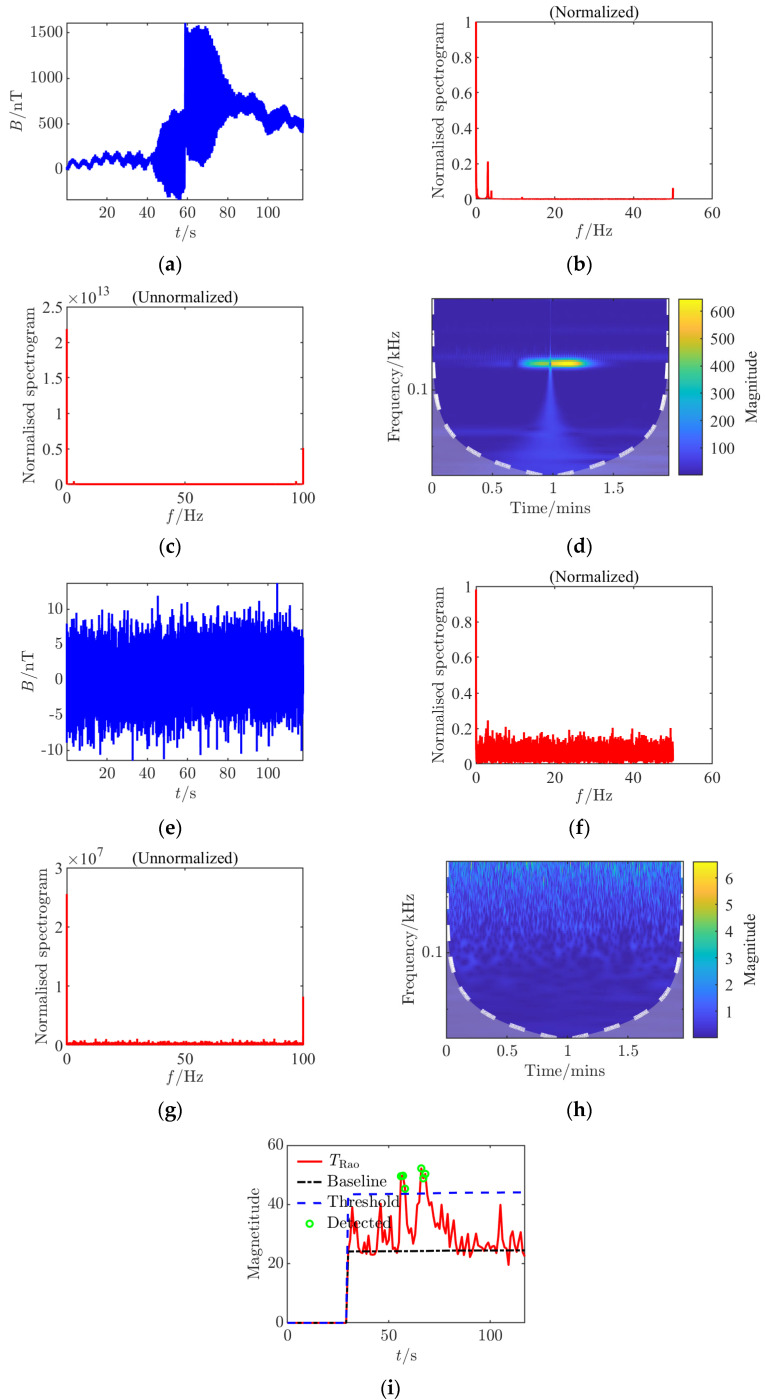
Time–frequency characteristics and detection results of Condition 4 at the signal detection threshold. (**a**) Time domain waveform; (**b**) normalized frequency spectrum of the clean signal; (**c**) raw frequency spectrum after noise removal; (**d**) continuous wavelet transform; (**e**) time domain waveform after noise addition; (**f**) normalized noisy frequency spectrum; (**g**) raw frequency spectrum with noise; (**h**) continuous wavelet transform after noise addition; (**i**) *Rao* test results.

**Figure 7:**
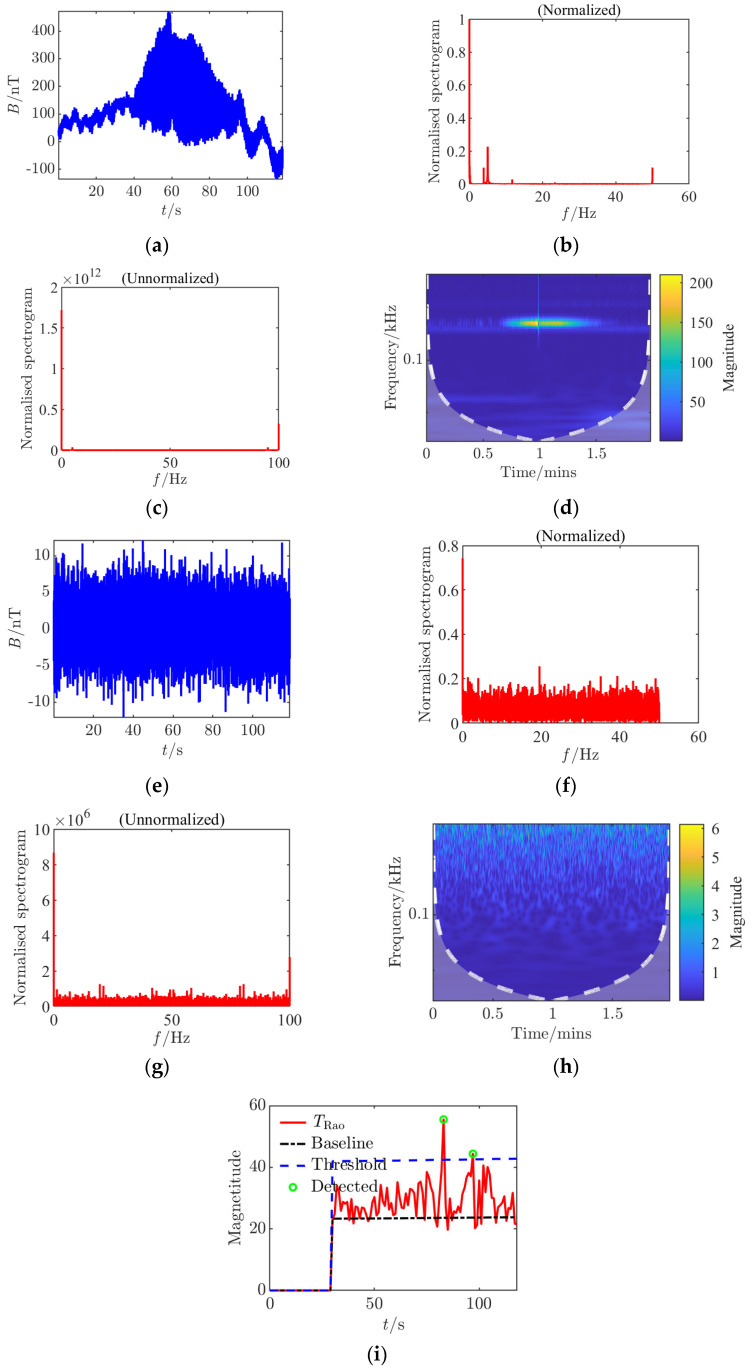
Time–frequency characteristics and detection results of Condition 5 at the signal detection threshold. (**a**) Time domain waveform; (**b**) normalized frequency spectrum of the clean signal; (**c**) raw frequency spectrum after noise removal; (**d**) continuous wavelet transform; (**e**) time domain waveform after noise addition; (**f**) normalized noisy frequency spectrum; (**g**) raw frequency spectrum with noise; (**h**) continuous wavelet transform after noise addition; (**i**) *Rao* test results.

**Figure 8:**
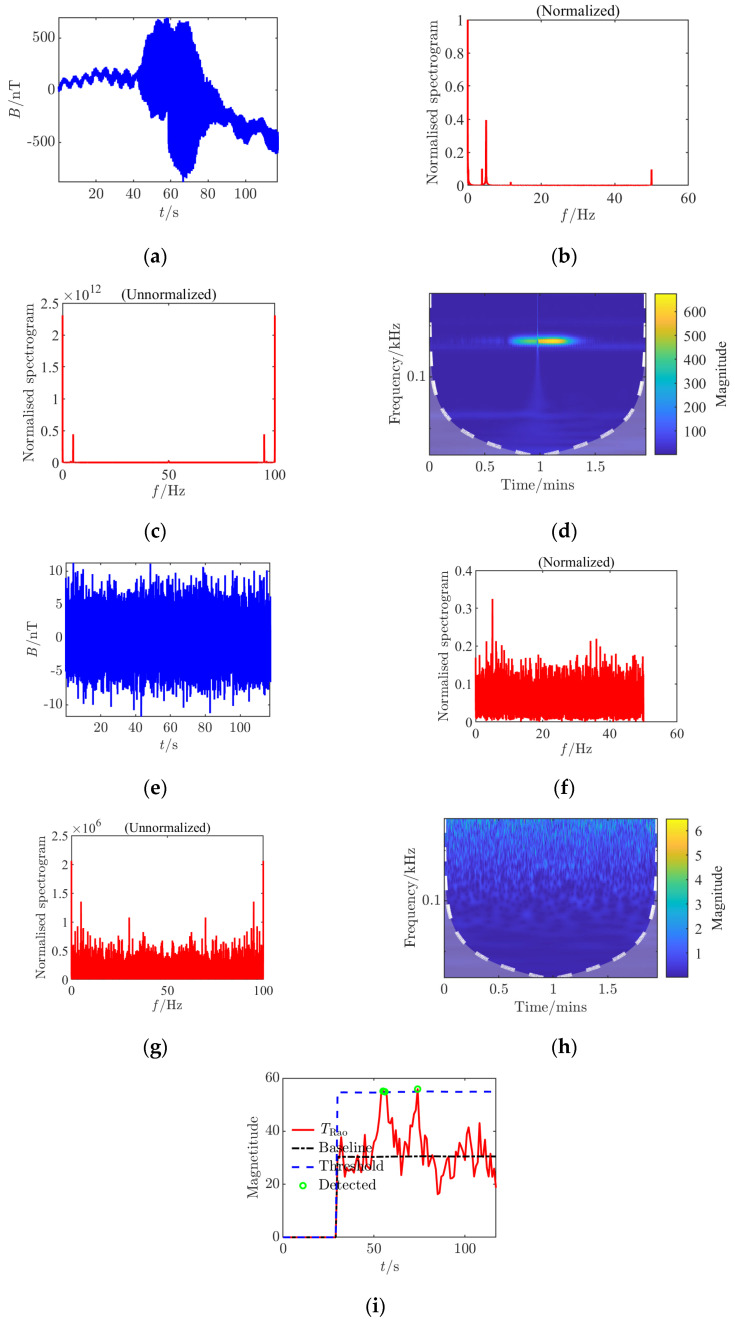
Time–frequency characteristics and detection results of Condition 6 at the signal detection threshold. (**a**) Time domain waveform; (**b**) normalized frequency spectrum of the clean signal; (**c**) raw frequency spectrum after noise removal; (**d**) continuous wavelet transform; (**e**) time domain waveform after noise addition; (**f**) normalized noisy frequency spectrum; (**g**) raw frequency spectrum with noise; (**h**) continuous wavelet transform after noise addition; (**i**) *Rao* test results.

For each set of collected data, processing and analysis based on the *Rao* detector were conducted, generating nine analysis charts including time-domain waveforms before and after adding noise, spectra before and after adding noise (normalized before and after), continuous wavelet transforms before and after adding noise, and *Rao* detection results. By comparing different CPA distances, it was found that at a 45 cm distance, the amplitude fluctuation of the signal in the time domain is smaller, the signal frequency components in the spectrum are less affected by noise, and the detection probability of the *Rao* detector is significantly higher compared to an 80 cm distance, with a noticeable improvement in the limit measurable SNR, indicating that a smaller CPA distance is more favorable for detection. This study innovatively constructed multi-dimensional time–frequency feature analysis during the experimental verification phase, implementing systematic signal analysis based on dynamic threshold *Rao* detectors for each set of experimental data. By simultaneously generating multi-dimensional feature maps, it achieved the first-time joint visualization of time–frequency-statistical three-dimensional features of shaft frequency magnetic field signals. These quantified data revealed for the first time the nonlinear mapping relationship between the dynamic threshold mechanism and propagation attenuation characteristics, providing key theoretical support for constructing a threshold adaptive compensation algorithm based on propagation distance in subsequent studies nT (see Figure 3, Figure 4, Figure 5, Figure 6, Figure 7 and Figure 8).

## 5. Conclusions

The experimental results show that the sliding threshold detection method based on *Rao* detector can effectively detect the axial magnetic field signal of electrical source under different frequencies and CPA distance conditions simulated in the laboratory, as shown in Table 1.

**Figure 9:**
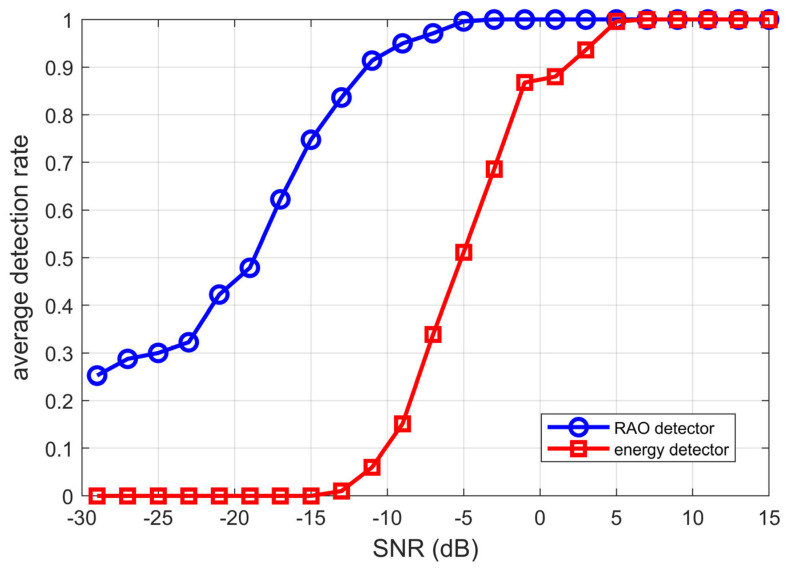
Detection probabilities of the *Rao* detector and conventional energy detector versus SNR.

The signal-to-noise ratio (SNR) varied from −30 dB to 15 dB in increments of 2 dB. For each SNR level, 1000 Monte Carlo simulations were conducted to evaluate the detection probabilities of the *Rao* detector and the conventional energy detector. The comparison of their detection performances is presented in Figure 9.

This paper proposes a joint time–frequency analysis method that combines *Rao* detector with dynamic sliding thresholds to enhance the detection performance of electric source axial frequency magnetic field signals under low signal-to-noise ratio conditions. For each signal-to-noise ratio point, 1000 independent Monte Carlo simulations were conducted, showing that this method achieves over 90% detection probability at approximately −13 dB SNR, significantly outperforming traditional energy detectors. Additionally, the impact of signal frequency and CPA distance on detection performance is analyzed, validating the algorithm’s robustness and practicality in complex non-Gaussian noise environments.

This work not only verifies the theoretical advantages of the *Rao* detector for low SNR axial frequency magnetic field signal detection but also establishes a quantitative correlation model between the sliding threshold mechanism and operational parameters, laying the key technical foundation for adaptive parameter configuration in intelligent detection systems. These findings provide important theoretical guidance for ship magnetic feature detection in actual marine environments: by constructing a frequency preference matrix and distance compensation algorithms, detection probability can be significantly improved in complex electromagnetic interference conditions.

In summary, the proposed method offers an effective technical solution for low SNR detection of ship axial frequency magnetic fields, demonstrating good application potential.

However, the method still has certain limitations. First, the algorithm’s computational complexity is high, which may pose challenges for real-time processing applications; second, this study mainly validates the method using simulation data, and further testing is needed for multi-source interference and non-ideal conditions in actual environments. Future work will focus on optimizing the algorithm’s real-time performance, combining more advanced signal modeling and filtering techniques to improve detection accuracy and stability in multi-interference environments, thereby further promoting the transition of this technology to practical engineering applications.

## Figures and Tables

**Table 1:** Statistics of detection under different working conditions.

Power Axis Frequency Magnetic Field Current (A)	Frequency (Hz)	CPA Distance (cm)	Number of Replication	Signal Detection Threshold (dB)
5	1	45	2	−17
		80	1	−15
	2	80	1	−18
	3	45	2	−19
		80	1	−20
	5	45	1	−19

## Data Availability

The datasets generated and/or analyzed during the current study are available from the corresponding author upon reasonable request.
